# Vitamin D analogs enhance the anticancer activity of 5-fluorouracil in an *in vivo* mouse colon cancer model

**DOI:** 10.1186/1471-2407-13-294

**Published:** 2013-06-18

**Authors:** Magdalena Milczarek, Mateusz Psurski, Andrzej Kutner, Joanna Wietrzyk

**Affiliations:** 1 Department of Experimental Oncology, Ludwik Hirszfeld Institute of Immunology and Experimental Therapy, Polish Academy of Sciences, R. Weigla St. 12, Wroclaw, 53-114, Poland; 2 Division of Medicinal Chemistry and Microbiology, Wroclaw University of Technology, Wroclaw, Poland; 3 Pharmaceutical Research Institute, L. Rydygiera St. 8, Warsaw, 01-793, Poland

**Keywords:** Vitamin D analogs, Combined treatment, 5-Fluorouracil, Capecitabine, Anticancer activity, Colon cancer

## Abstract

**Background:**

Active vitamin D analogs that are less toxic than calcitriol can be useful in the combined treatment of patients suffering from colon cancer. In the present study we demonstrate, for the first time in an *in vivo* model system, the biological effect of combined therapy using 5-fluorouracil (5-FU) along with vitamin D analog PRI-2191 (tacalcitol, 1,24-dihydroxyvitamin D_3_) or PRI-2205 (5,6-trans-isomer of calcipotriol) on colon cancer.

**Methods:**

We investigated the influence of vitamin D analogs on the anticancer activity of 5-FU or capecitabine in the treatment of mice bearing MC38 mouse colon tumors implanted subcutaneously or orthotopically. The cell cycle distribution, E-cadherin expression and caspase 3/7 activity *in vitro* were also evaluated.

**Results:**

We observed that both PRI-2191 and PRI-2205 significantly enhanced the antitumor activity of 5-FU; but these results depend on the treatment regimen. Applying the optimal schedule of combined therapy we observed a significant decrease in tumor growth, metastasis and also a prolongation of the survival time of mice, in comparison with the administrations of 5-FU given alone. Both combinations indicated a synergistic effect and did not cause toxicity. Moreover, analogs applied after completed course of administration of 5-FU, prolonged the antitumor effect of the drug. Furthermore, when the prodrug of 5-FU, capecitabine, was used, potentiation of its activity was also observed.

**Conclusions:**

Our data suggest that vitamin D analogs (especially PRI-2191) might be potentially applied to clinical use in order to enhance the anticancer effect of 5-FU and also prolong its activity against colon cancer. The activity of PRI-2191 is realized through stopping the cells in the G_0_/G_1_ cell cycle phase and increasing the expression of E-cadherin.

## Background

According to the World Health Organization’s International Agency for Research on Cancer, colorectal cancer is the third most frequent malignancy and the fourth leading cause of deaths from cancer worldwide [1]. Despite significant progress in the treatment of patients suffering from colorectal cancer in the last decade, there is a constant need for new therapies. One of the directions is the development of novel combined treatment strategies. The benefit of such an approach is the possibility of enhancing the therapeutic effect of a drug, which is the basis of a standard therapy. Promising candidates for this strategy are vitamin D analogs.

Epidemiological and clinical data and also research on animals suggest a protective role for the active form of vitamin D (calcitriol, 1,25-dihydroxyvitamin D_3_) in the development of colon cancer [2-4]. Data from the *in vivo* studies have shown that a diet supplemented with vitamin D significantly delayed MC-26 colon cancer tumor growth compared to a diet deficient in this vitamin [5]. Calcitriol affects proliferation, differentiation and apoptosis of human colon cancer cells. It exerts a biological effect mainly through the vitamin D receptor (VDR) [6]. It has been shown that the expression of VDR increases from normal colon epithelial cells through precancerous lesions to well-differentiated tumors and then decreases in advanced stages of cancer [6,7].

The antitumor activity of calcitriol is observed only when it is applied in hyper-physiological doses, which can cause the side effect of hypercalcemia and hypercalciuria [8-10]. For this reason, the synthesis of analogs has been initiated in order to dissociate the calcemic effect from the anticancer activity of calcitriol. In our previous studies, we have examined the biological activity of a series of side-chain modified analogs of vitamin D and a series of diastereometric and geometric ones against various cancer and normal cell lines [11,12]. We also evaluated the influence of vitamin D analogs on the activity of a range of anticancer drugs *in vitro* and *in vivo* against the human and murine cancer cells [13-18]. We observed that vitamin D analogs increased the antitumor effect of cyclophosphamide and cisplatin compared to the cytostatic drug applied alone. Based on our results, we selected two analogs for further research: PRI-2191 (tacalcitol, 1,24-dihydroxyvitamin D_3_) and PRI-2205 (5, 6-trans calcipotriol), which reveal higher antitumor activity and lower calcemic activity, as well as lower toxicity than calcitriol [12,19]. These two analogs, used in combined HT-29 colon cancer treatment with irinotecan or oxaliplatin showed, in selected schedules of treatment, improvement in mice survival and tumor growth delay [20].

5-Fluorouracil (5-FU) is one of the oldest anticancer drugs and is still used in the treatment of colorectal cancer [21,22]. Two recent reports of *in vitro* studies demonstrate that calcitriol and calcipotriol promote the sensitivity of human colon carcinoma cells to 5-FU and enhance the cytotoxicity of the FOLFIRI anticancer regimen. These results also indicate that the mechanism of calcitriol and calcipotriol action is dependent on the calcium sensing receptor (CaSR). Protein expression and the gene transcriptional activity of survivin and thymidylate synthase are suppressed by inducing the expression and activation of CaSR by calcitriol or calcipotriol. This leads to an increase in the sensitivity of colon carcinoma cells to 5-FU [23,24].

Therefore, the aim of our present studies was to examine the biological effect (antitumor activity, influence on the life span of mice, toxicity and antimetastatic activity) of combined therapy with the use of 5-FU along with PRI-2191 or PRI-2205 against MC38 mouse colon cancer *in vivo.* In addition, we examined whether vitamin D analogs would prolong the antitumor activity of 5-FU (application of analogs was initiated after administration of 5-FU ended).

## Methods

### Compounds

Calcitriol and its analogs: PRI-2191, PRI-2201 and PRI-2205 are certified synthetic materials obtained from the Pharmaceutical Research Institute, Warsaw, Poland. Samples of the compounds were stored, under argon, in amber ampoules at -20°C. Prior to usage, in the case of *in vitro* studies, compounds were dissolved in 99.8% ethanol to the concentration of 10^-4^ M and subsequently diluted in culture medium in order to reach the concentration of 100 nM. For animal experiments, compounds were dissolved in 99.8% ethanol, then diluted in 80% propylene glycol in order to reach the required concentrations and administered subcutaneously (s.c.) or orally (p.o.) to mice in a volume of 5 μl per 1 g of body weight.

5-Fluorouracil (5-FU) (Ebewe Pharma, Unterach, Austria) solution in the concentration of 50 mg/ml was diluted prior to usage in *in vitro* studies in culture medium in order to reach the required concentrations and for *in vivo* experiments in saline in order to reach the required concentrations and administered either intravenously (i.v.) or intraperitoneally (i.p.) to mice at a volume of 10 μl per 1 g of body weight.

Capecitabine (CPC) (Pharmaceutical Research Institute, Warsaw, Poland) was dissolved in 40% ethanol, then diluted in water for injection in order to reach the require concentration and administered orally (p.o.) to mice at a volume of 10 μl per 1 g of body weight.

### Cell lines

The mouse colon adenocarcinoma cell line MC38, cultured *in vivo*, was obtained from the Tumor Bank of the TNO Radiobiology Institute, Rijswijk, Holland. This cell line was adapted to growth *in vitro* as MC38/0 [25]. The MC38/EGFP mouse colon cancer cells, transduced with green fluorescent protein gene and cultured *in vitro,* were obtained from the Institute of Immunology and Experimental Therapy, Wroclaw, Poland [25]. Human colon adenocarcinoma cell lines HT-29 and LoVo were received from the Deutsches Krebsforschungszentrum, Heidelberg, Germany. All cell lines were stored in liquid nitrogen at the Cell Culture Collection of the Institute of Immunology and Experimental Therapy, Wroclaw, Poland.

The cell lines were cultured *in vitro* as follows: MC38/0 and MC38/EGFP in RPMI 1640 medium (IIET, Wroclaw, Poland), HT-29 and LoVo in RPMI 1640 + Opti-MEM I (1:1) (from IIET, Wroclaw, Poland and Gibco, Scotland, UK, respectively) all culture media were supplemented with 2 mM L-glutamine, 1 mM sodium pyruvate (both from Sigma-Aldrich Chemie GmbH, Steinheim, Germany), 5% fetal bovine serum (PAA Laboratories GmbH, Pasching, Austria (MC38/0 and MC38/EGFP) or Thermo Fisher Scientific Inc., UK (HT-29 and LoVo) and 100 U/ml penicillin, 100 μg/ml streptomycin (both from Polfa Tarchomin S.A. Warsaw, Poland). The cells were cultured at 37°C in a humid atmosphere saturated with 5% CO_2_.

### Mice

C57BL/6 female, 12-16-week-old mice, weighing 20–25 g were obtained from the Maria Sklodowska-Curie Institute – Oncology Center (Warsaw, Poland) and maintained under specific pathogen-free (SPF) conditions. All experiments were performed according to EU Directive 2010/63/EU for animal experiments and were approved by the 1^st^ Local Committee for Experiments with the Use of Laboratory Animals, Wroclaw, Poland.

### Details of the treatment schedules

The MC38 colon cancer cells were passaged *in vivo*. *Subcutaneous transplantation:* mice were subcutaneously (s.c.) inoculated in the right flank region with a 33% suspension of homogenized MC38 tumor tissue coming from s.c. tumors from another mouse, 0.25 ml per mouse. *Orthotropic transplantation:* the anesthetized mouse was placed on a wooden board in the right lateral position and the incision was made through the left upper abdominal pararectal line and peritoneum. The cecal wall was carefully exposed, placed and fixed between layers of sterile gauze. 1 – 2 mm piece of specimen, derived from primary tumor grown s.c. in another mouse, was fixed to the serosal part of cecal wall with 5–0 surgical sutures. After implantation, the peritoneum and abdominal wall was sutured with 4–0 surgical sutures (Dexon-“S”, Polfa, Poznań, Poland). Tumor cell transplantations were performed under general anesthesia with the mixture of ketamine hydrochloride (100 mg/kg, Ketamina 10%, Biowet, Puławy, Poland) and xylazine hydrochloride (20 mg/kg, XylaRiem, Riemser Arzneimittel AG, Germany).

The MC38/EGFP colon cancer cells derived from *in vitro* culture were inoculated s.c. in the right flank region with 1 × 10^6^ cells suspended in 0.2 ml saline per mouse. After tumor inoculation, mice were randomly divided into different groups (day 0).

#### *The evaluation, both the most effective dose and treatment schedule of vitamin D analogs in combination with 5-FU*

*Vitamin D analog was administered s.c. three times a week.* In these experiments, different doses of vitamin D analogs and varied doses and treatment schedules of 5-FU were studied (Table 1 – 5-FU and PRI-2191, Table 2 – 5-FU and PRI-2205). In each experiment, mice bearing s.c. MC38 colon cancer cells were randomly divided into different groups (day 0). Then, the mice were injected with 5-FU and/or vitamin D analogs.

**Table 1:** The effect of vitamin D analog PRI-2191 (in varied doses) alone or in combination with 5-FU on tumor growth, life span and body weight of mice bearing subcutaneous colon cancer MC38

**No.**	**5-FU**	**PRI-2191**	**TGI [%]**					**ILS [%]**					**Maximal decrease**		
													**in body weight [%]**		
			**5-FU**	**PRI-2191**	**5-FU+**	**%H TGI**	**Effect**	**5-FU**	**PRI-2191**	**5-FU+**	**%H ILS**	**Effect**	**5-FU**	**PRI-2191**	**5-FU+**
					**PRI-2191**					**PRI-2191**					**PRI-2191**
1	75 mg/kg;	2 μg/kg	48	6	70^*^	51	Synergism	21	53	41	62	Antagonism	7	18	19
2	150 mg/kg;	2 μg/kg	87	44	96^*^	92	Synergism	nt.	nt.	nt.	nt.	nt.	9	11	21
3	100 mg/kg;	1 μg/kg	38	6	51	42	Synergism	26	-6	118^*a^	22	Synergism	4	6	12
4	100 mg/kg;	0.5 μg/kg	38	1	36	39	Antagonism	26	-6	43	22	Synergism	4	3	9
5	100 mg/kg;	0.25 μg/kg	38	-35	36	16	Antagonism	26	3	65	29	Synergism	4	9	9

Vitamin D analog was administered subcutaneously, three times a week.

No. – number of experiment; TGI – tumor growth inhibition; %H TGI: hypothetical tumor growth inhibition; ILS: increase in life span; %H ILS: hypothetical increase in life span; nt.: not tested; No. 1 – TGI calculated on the 27th day of the experiment, No. 2, 3, 4, 5 – TGI on the 32nd day of the experiment.

No. 1: 5-FU was injected i.p. on the first day; PRI-2191 was injected on days: 1, 3, 6, 8, 10, 13, 15, 17, 20, 22; Number of mice: control - 10; in the each treatment group - 8.

No. 2: 5-FU was injected i.p. on days: 9, 16, 23; PRI-2191 was injected on days: 11, 14, 16, 18, 21, 23; Number of mice in each group - 8.

No. 3, 4 and 5: 5-FU was injected i.v. on days: 8, 15, 30, 38; PRI-2191 was injected on days: 9, 11, 14, 16, 18, 21, 23, 25, 28, 30, 32, 35, 37, 39, 42, 44; Number of mice: No. 3, 4 and 5: control – 7; 5-FU – 7, PRI-2191 – 5; 5-FU + PRI-2191 – 6; ^*^P < 0.05 as compared to control; ^*a^*P* < 0.05 as compared to 5-FU.

**Table 2:** The effect of vitamin D analog PRI-2205 (in varied doses) alone or in combination with 5-FU on tumor growth, life span and body weight of mice bearing subcutaneous colon cancer MC38

**No.**	**5-FU**	**PRI-2205**	**TGI [%]**					**ILS [%]**					**Maximal decrease**		
													**in body weight [%]**		
			**5-FU**	**PRI-2205**	**5-FU+**	**%H TGI**	**Effect**	**5-FU**	**PRI-2205**	**5-FU+**	**%H ILS**	**Effect**	**5-FU**	**PRI-2205**	**5-FU+**
					**PRI-2205**					**PRI-2205**					**PRI-2205**
1	75 mg/kg;	10 μg/kg	48	7	76^*^	44	Synergism	21	2	77^*a^	23	Synergism	7	10	8
2	50 mg/kg;	10 μg/kg	30	-16	70	19	Synergism	12	0	38^*^	12	Synergism	0	0	0
3	150 mg/kg;	10 μg/kg	87	25	96^*^	90	Synergism	nt.	nt.	nt.	nt.	nt.	9	1	19
4	100 mg/kg;	5 μg/kg	31	-12	26	23	Antagonism	26	-6	12	22	Antagonism	4	1	6
5	100 mg/kg;	2.5 μg/kg	31	-41	19	2	Antagonism	26	-15	32	16	Synergism	4	7	4

Vitamin D analog was administered subcutaneously, three times a week.

No. – number of experiment; TGI – tumor growth inhibition; %H TGI: hypothetical tumor growth inhibition; ILS: increase in life span; %H ILS: hypothetical increase in life span; nt.: not tested; No. 1 – TGI calculated on the 27th day of the experiment, No. 2, 4, 5 - TGI on the 30th day of the experiment; No. 3 – TGI on the 32nd day of the experiment.

No. 1: 5-FU was injected i.p. on the first day; PRI-2205 was injected on days: 1, 3, 6, 8, 10, 13, 15, 17, 20, 22; Number of mice: control - 10; in the each treatment group - 8.

No. 2: 5-FU was injected i.p. on days: 2, 7, 11, 16; PRI-2205 was injected on days: 2, 4, 7, 9, 11, 14, 16, 18, 21; Number of mice: control – 9, 5-FU – 8, 5-FU + PRI-2205 – 8, PRI-2205 – 7.

No. 3: 5-FU was injected i.p. on days: 9, 16, 23; PRI-2205 was injected on days: 11, 14, 16, 18, 21, 23, 25, 28; Number of mice in each group - 8.

No. 4 and 5: 5-FU was injected i.v. on days: 8, 15, 30, 38; PRI-2205 on days: 9, 11, 14, 16, 18, 21, 23, 25, 28, 30, 32, 35, 37, 39, 42, 44; Number of mice: No. 4 and 5: control – 7, 5-FU – 7, PRI-2205 – 5, 5-FU + PRI-2205 – 6; ^*^P < 0.05 as compared to control; ^*a^*P* < 0.05 as compared to 5-FU.

*Vitamin D analog was administered s.c. five times a week.* Mice bearing s.c. MC38 colon cancer cells were i.v. injected with 5-FU at a dose of 100 mg/kg/day on days: 2, 17 and/or vitamin D analogs: PRI-2191 at a dose of 0.2 μg/kg/day or PRI-2205 at a dose of 20 μg/kg/day. Both analogs were administrated s.c., five times a week on days: 9, 10, 11, 14, 15, 16, 17, 18, 21, 22, 23, 24, 25.

*Vitamin D analog was administered p.o. three times a week.* Mice bearing s.c. MC38 colon cancer cells were i.v. injected with 5-FU at a dose of 100 mg/kg/day on days: 9, 16, 23 and/or vitamin D analogs: PRI-2191 at a dose of 1 μg/kg/day or PRI-2205 at a dose of 10 μg/kg/day. Both analogs were administrated p.o. by gavage, directly into the lower esophagus using a feeding needle, three times a week on days: 12, 14, 16, 19, 21, 23, 26, 28.

#### *The effect of combined therapy with 5-FU and PRI-2191 or PRI-2205 on MC38 colon tumor growth implanted orthotopically*

Mice bearing MC38 tumors implanted i.i. were i.v. injected with 5-FU at a dose of 100 mg/kg/day on days: 8, 15 and/or vitamin D analogs: PRI-2191 at a dose of 1 μg/kg/day or PRI-2205 at a dose of 10 μg/kg/day. Both analogs were administrated s.c., three times a week on days: 10, 13, 15, 17, 20. Mice were sacrificed on day 22 after i.i. transplantation, tumors were weighed and blood was collected.

#### *The prolongation of 5-FU antitumor activity by PRI-2191 or PRI-2205*

Mice bearing s.c. MC38 colon cancer cells were i.v. injected with 5-FU at a dose of 100 mg/kg/day on days: 20, 24, 28, 34. When 5-FU reduced tumor volume by about 90% in comparison to the control group, the administration of analogs was initiated (after administration of the cytostatic drug ended). Both analogs were administrated s.c., three times a week on days: 35, 38, 40, 42, 45, 47, 49, 52, 54, 56, PRI-2191 at a dose of 1 μg/kg/day and PRI-2205 at a dose of 10 μg/kg/day.

#### *The antimetastatic effect of combined therapy with 5-FU and PRI-2191 or PRI-2205*

Mice bearing s.c. MC38/EGFP colon cancer cells were i.p. treated with 5-FU at a dose of 75 mg/kg/day on days: 19, 24, 29 and/or vitamin D analog PRI-2191 at a dose of 1 μg/kg/day or PRI-2205 at a dose of 10 μg/kg/day. Both analogs were administrated s.c., three times a week on days: 19, 21, 24, 26, 28, 31, 33, 35, 38, 40, 42, 45, 47, 49, 52, 54, 56. On the 59th day of the experiment the lymph nodes were isolated and then observed under a NightOWL II LB 983 device at Wroclaw University of Environmental and Life Sciences, Poland, in order to visualize and calculate the metastasis of s.c. tumor cells to regional lymph nodes, liver, spleen and lung. Only the metastases of MC38/EGFP colon cancer cells to the lymph nodes were observed.

#### *The effect of combined therapy with capecitabine and PRI-2191 or PRI-2205 on MC38 colon tumor growth implanted subcutaneously*

Mice bearing MC38 tumors implanted s.c. were p.o. (by gavage, directly into the lower esophagus using a feeding needle) administered with capecitabine at a dose of 450 mg/kg/day on days: 3, 4, 5, 6, 7, 10, 11, 12, 13, 14 and/or vitamin D analogs: PRI-2191 at a dose of 1 μg/kg/day or PRI-2205 at a dose of 10 μg/kg/day. Both analogs were administrated s.c., three times a week on days: 3, 5, 7, 10, 12, 14, 17, 19, 21, 24, 26, 28, 31, 33, 35, 38, 40, 42, 45, 47, 49, 52, 54.

### Evaluation of the therapeutic effect

Tumor volume was calculated using the formula (*a*^2^ × *b*)/2, where *a* = shorter tumor diameter in mm and *b* = longer tumor diameter in mm. Inhibition of tumor growth was calculated from the following formula: tumor growth inhibition (TGI) [%] = 100 – [(W_T_/W_C_) × 100], where W_T_ is the median tumor weight of treated mice and W_C_ – that of untreated control mice. Mice obviously sick were sacrificed and the increase in life span (ILS) of treated mice over the control was evaluated. ILS was calculated from the following formula: 100 – [(MST_T_/MST_C_) × 100], where MST_T_ is the median survival time of treated animals, and MST_C_ is the median survival time of untreated control mice.

#### *Evaluation of combination effects*

The TGI and ILS values were then compared with hypothetical tumor growth inhibition: %H TGI or %H ILS = 100 - [(100 - E for cytostatic) × (100 - E for vitamin D analog) /100] [26], where E was TGI or ILS.

As the result of the comparison of TGI to TGI hypothetical value (%H TGI) or ILS to ILS hypothetical value (%H ILS) the type of interaction between two compounds in combined treatment was designated, which can be:

•synergy – when the experimental value of TGI/ILS is greater than hypothetical TGI/ILS;

•additive effect – when those two values are comparable;

•subadditive effect – if the experimental TGI/ILS value is smaller than the hypothetical, but larger than TGI/ILS for cytostatic given alone;

•antagonism – if the experimental TGI/ILS is smaller than the experimental TGI/ILS for cytostatic.

#### *Tumor growth delay*

Tumor growth delay (TGD) is a parameter describing the time needed for tumors to reach the volume of 1 cm^3^. It can be also expressed as the direct difference in days: ΔTGD_control_ = TGD _treated group_–TGD_control_, where TGD_treated group_ are the days needed to reach the volume of 1 cm^3^ for tumors in the treated group and TGD_control_ the days needed for tumors to reach the volume of 1 cm^3^ in control group. In the case of combined treatment: ΔTGD_cytostatic_ = TGD_treated group_–TGD_cytostatic_, where TGD_cytostatic_ are the days needed for tumors to reach the volume of 1 cm^3^ in the treated group with cytostatic drugs.

#### *Body weight changes*

The average body weight change (BWC) in all groups was calculated using the formula: BWC = (ABW_n_/ABW_1_) × 100 – 100%, where ABW_*n*_ is the average body weight on the nth-day of experiment (during treatment) and ABW_1_ is the average body weight on the first day of treatment.

#### *Blood leukocytes and platelets*

The level of blood leukocytes and platelets was measured in each individual blood sample (Sysmex K4500SL, serial number F2872, Japan).

#### *Serum calcium level*

Female BALB/c mice were treated i.p. with 5-FU at a dose of 50 and 75 mg/kg on days 1 and 8, respectively and/or with vitamin D analogs: PRI-2191 at a dose of 2 μg/kg/day or PRI-2205 at a dose of 10 μg/kg/day. Both analogs were administrated s.c., three times a week on days: 1, 3, 6, 8, 10, 13. Mice were sacrificed 16 days after the first injection of the compounds and blood sera were collected. The calcium level was measured in each individual serum sample with the photometric Arsezano 3 method (Olympus AU400; Olympus America Inc., Melville, NY, USA).

### An anti-proliferative assay *in vitro*

24 hours before the addition of the tested compounds, cells were plated in 96-well plates (Sarstedt, Newton, NC, USA) at a density of 2 × 10^4^ cells per well. The cells were exposed for 120 h to 100 nM of calcitriol, PRI-2191, PRI-2205 or PRI-2201 and simultaneously to 5-FU: 0.1 μg/ml (HT-29 and LoVo) or 0.01 μg/ml (MC38/0); and the SRB assay for evaluating the cytostatic effect was performed as described previously [12]. The results were calculated as the proliferation inhibition of the cancer cell population. Each compound was tested four-fold either alone or in combined treatment in a single experiment, which was repeated 3–5 times.

The percentage of proliferation inhibition was calculated according to the formula: % of proliferation inhibition = [(At-Am)/(Ac-Am)*100]-100; where: At – absorbance of treated cells; Am – absorbance of culture medium; Ac – absorbance of control cells. The value of the percentage of proliferation inhibition was then compared with the hypothetical percentage of proliferation inhibition: %H = 100-[(100-E for cytostatic) x (100-E for calcitriol analog) /100], where E was the mean value of the percentage of proliferation inhibition in order to determine the interaction between two compounds in combined treatment [26]. The type of interaction was described above.

### Cell cycle analysis

Cultured HT-29 cells were seeded at a density of 7.5 × 10^3^ cells/ml of culture medium on 6-well plates (Corning, NY, USA) to a final volume of 4 ml. The cells were exposed to compounds at set concentrations for 120 h: PRI-2191 and PRI-2205 100 nM; 5-FU 0.1 μg/ml. Ethanol, used as a solvent for all compounds, diluted corresponding to its highest concentration, produced no toxicity when applied to the compounds. After 120 h of incubation, the cells were collected with the use of trypsin/EDTA, pH 8 (IIET, Wroclaw, Poland), washed in phosphate-buffered saline (PBS) supplemented with 2% of fetal bovine serum and counted in a hemacytometer. Cells (1 × 10^6^ per sample) were then washed twice in cold phosphate buffered saline (PBS) and fixed for 24 h in 70% ethanol at -20°C. The cells were then washed twice in PBS and incubated with RNAse (8 μg/ml, Fermentas GmbH, St. Leon-Rot, Germany) at 37°C for 1 h. The cells were stained for 30 min with propidium iodide (0.5 mg/ml; Sigma-Aldrich Chemie GmbH, Steinheim, Germany) at 4°C and the cellular DNA content was determined using a BD FACSCalibur instrument (Becton Dickinson, San Jose, CA, USA) and a ModFit LT 3.0 program (Verity Software House, Topsham, ME, USA). The experiment was repeated 4 times.

### E-cadherin expression

Cultured HT-29 cells were seeded at a density of 1 × 10^5^ cells/ml of culture medium on 6-well plates (Corning, NY, USA) to a final volume of 4 ml. The cells were exposed to compounds at set concentrations for 48 h: PRI-2191 and PRI-2205 100 nM; 5-FU 0.1 μg/ml. After 48 h of incubation, the cells were collected using a non-enzymatic cell dissociation solution (Sigma-Aldrich Chemie GmbH, Steinheim, Germany), washed in phosphate-buffered saline supplemented with 1% of fetal bovine serum (1% FBS) and counted in a hemacytometer. The cells (2 × 10^5^ per sample) were washed once with 1% FBS and then suspended in 0.1 ml of 5% bovine serum albumin solution in PBS (Sigma-Aldrich Chemie GmbH, Steinheim, Germany) and incubated for 20 min at room temperature. After incubation, the cells were washed with 1% FBS and then were stained with anti-E-cadherin conjugated with phycoerythrin (PE) or anti-IgG_1_-PE (both from Abcam, Cambridge, UK) for 30 min in the dark at room temperature. After incubation, the cells were washed with 1% FBS and then suspended in 0.3 ml in the same solution. Data analysis was performed by flow cytometry using a BD LSRFortessa instrument (Becton Dickinson, San Jose, CA, USA). Next, data were analysed in a BD FACSDiva 6.2 program. The experiment was repeated 3 times.

### Active caspase-3/7 and cell death (subG_1_) analysis

Cultured MC38/0 cells were seeded at a density of 1 × 10^5^ cells/ml of culture medium on 24-well plates for caspase-3/7 activity assay or 6-well plates for subG_1_ stage analysis (both from Corning, NY, USA) to a final volume of 2 ml or 4 ml, respectively. The cells were exposed to compounds at set concentrations for 48 h: PRI-2191 and PRI-2205 100 nM; 5-FU 50 and 200 μg/ml. Ethanol, used as a solvent for all compounds and diluted corresponding to its highest concentration, produced no toxicity when applied to the compounds.

#### *Enzymatic caspase-3/7 activity assay*

The experiment was based on a previously described method [27] adapted to cell-based assay. After treatment, MC-38/0 cell were lysed using ice-cold lysis buffer (50 mM HEPES, 10% (w/v) sucrose, 150 mM NaCl, 2 mM EDTA, 1% (v/v) Triton X-100, pH 7.3) (IIET, Wroclaw, Poland). After 10 min., 40 μl of each sample was transferred to a white, 96-well plate (Corning, NY, USA) containing 160 μl of reaction buffer (20 mM HEPES, 10% (w/v) sucrose, 100 mM NaCl, 1 mM EDTA, 10 mM DTT, 0.02% (v/v) Trition X-100, pH 7.3) (IIET, Wroclaw, Poland) with 9 μM Ac-DEVD-ACC (λ_ex_ = 360 nm, λ_em_ = 460 nm) as a fluorogenic substrate. Fluorescence increase correlated with caspase-3/7 level was continuously recorded at 37°C for 90 min using a Biotek Synergy H4 (Biokom, Warsaw, Poland). Results were normalized to the protein content determined using the Lowry method (BioRad, Warsaw, Poland) and are reported as relative caspase-3/7 activity in comparison to the untreated control.

#### *Death cell analysis with propidium iodide staining (subG1 stage)*

After 48 h of incubation the cells were prepared in the same manner as cells for the assay of cell cycle distribution described above. Next, data analysis was performed by flow cytometry using a BD LSRFortessa instrument (Becton Dickinson, San Jose, CA, USA). Next, data were analysed in a BD FACSDiva 6.2 program. The experiment was repeated 4 times.

### Statistical evaluation

Statistical analysis of *in vivo* results was performed by employing STATISTICA version 7.1 (StatSoft, Inc., USA). For tumor growth inhibition analysis and for antimetastatic effect analysis the Kruskal-Wallis ANOVA Multiple Comparison *P* value (2-tailed) test was used. For survival analysis, the Peto & Peto modification of the Gehan-Wilcoxon test was used. The results of *in vitro* studies were analysed by employing STATISTICA version 10 (StatSoft, Inc., USA) using the Mann–Whitney U test for cell cycle, E-cadherin expression and death cell (subG_1_) analysis as well as the Tukey HSD test for caspase-3/7 activity assay. *P* values less than 0.05 were considered significant.

## Results

### The evaluation of both the most effective dose and treatment schedule of vitamin D analogs in combination with 5-FU

The analogs were injected to mice subcutaneously (s.c.) or orally (p.o.) three or five times a week. We also tested varied doses of 5-FU, which were administrated once or repeatedly, intraperitoneally (i.p.) or intravenously (i.v.) to establish the best scheme of the combined treatment of mice with MC38 colon cancer.

*Analog PRI-2191 or PRI-2205 administered s.c., three times a week.* The analog PRI-2191 was used in the following doses: 2, 1, 0.5 and 0.25 μg/kg/day. Combined treatment with 5-FU and PRI-2191 (2 μg/kg/day) significantly retarded the tumor growth when compared to the untreated mice. However, the analysis of ILS indicated an antagonism between both agents. A significant decrease was observed in the body weight of mice which received PRI-2191 alone or in combination with 5-FU (Table 1, No. 1, 2).

The application of lower doses of PRI-2191 (0.5 or 0.25 μg/kg/day) did not increase the tumor growth inhibition caused by 5-FU (in 38% in relation to untreated mice) (No. 4, 5). On the other hand, treatment with 5-FU and PRI-2191 prolonged the survival of mice by 43% (0.5 μg/kg/day) and 65% (0.25 μg/kg/day), while for 5-FU alone by 26%, but not in a statistically significant manner (Table 1, No. 4, 5).

**Figure 1:**
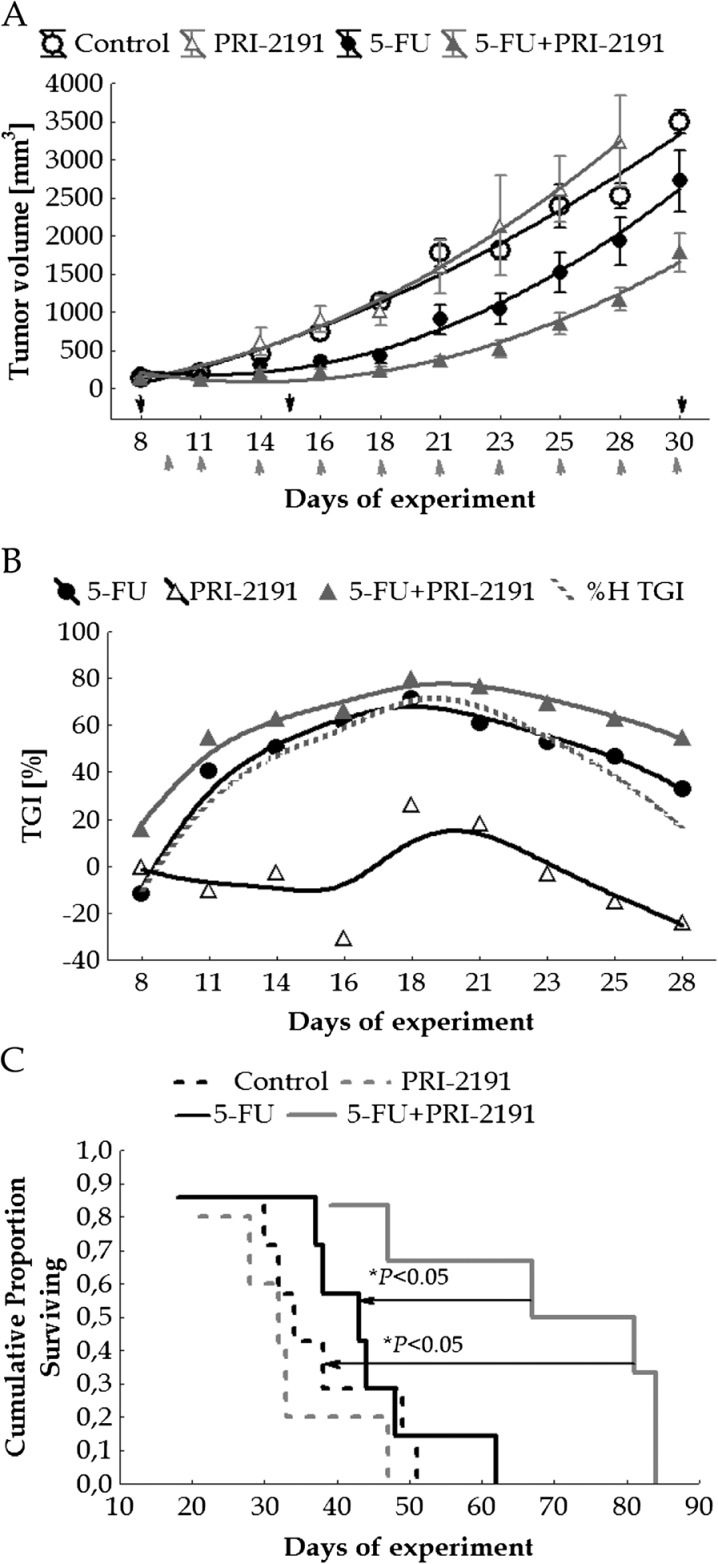
**The influence of PRI-2191 on the antitumor activity of 5-FU in MC38 colon cancer.** PRI-2191 was administered at a dose of 1 μg/kg/day, s.c., three times a week, from days 9 to 44, 5-FU at a dose of 100 mg/kg/day, i.v., from days 8 to 38. Black arrows indicate the days of 5-FU administration, gray arrows – days of PRI-2191 administration. **A**) Kinetics of tumor growth (mean of tumor volume). *P* < 0.05: 5-FU combined with PRI-2191 in comparison to the control from days 18 to 25 (Kruskal-Wallis ANOVA Multiple Comparisons *P* values (2-tailed) test). **B**) Tumor growth inhibition (TGI) and hypothetical TGI values were calculated from days 8 to 32 of the experiment (median of tumor volume). **C**) Survival analysis of treated and untreated mice. Number of mice: control – 7; 5-FU – 7, PRI-2191 – 5; 5-FU + PRI-2191 – 6.

Our results indicate that the best effective dose for analog PRI-2191 is 1 μg/kg/day in combined treatment with 5-FU (Table 1, No. 3, Figure 1). In this case, the analysis of TGI (Figure 1B) as well as ILS (Table 1, No. 3) indicated synergy between 5-FU and PRI-2191. A statistically significant prolongation of the survival of mice treated with both agents (118%) was observed in comparison with 5-FU alone (26%), as well as with untreated mice (Table 1, No. 3; Figure 1C). The combined treatment retarded tumor growth from the 8th day to the end of the experiment as compared to 5-FU treatment alone (in a statistically significant manner from the 18th to 25th day compared to untreated mice) (Figure 1A). The maximal decrease in body weight did not exceed 12%.

**Figure 2:**
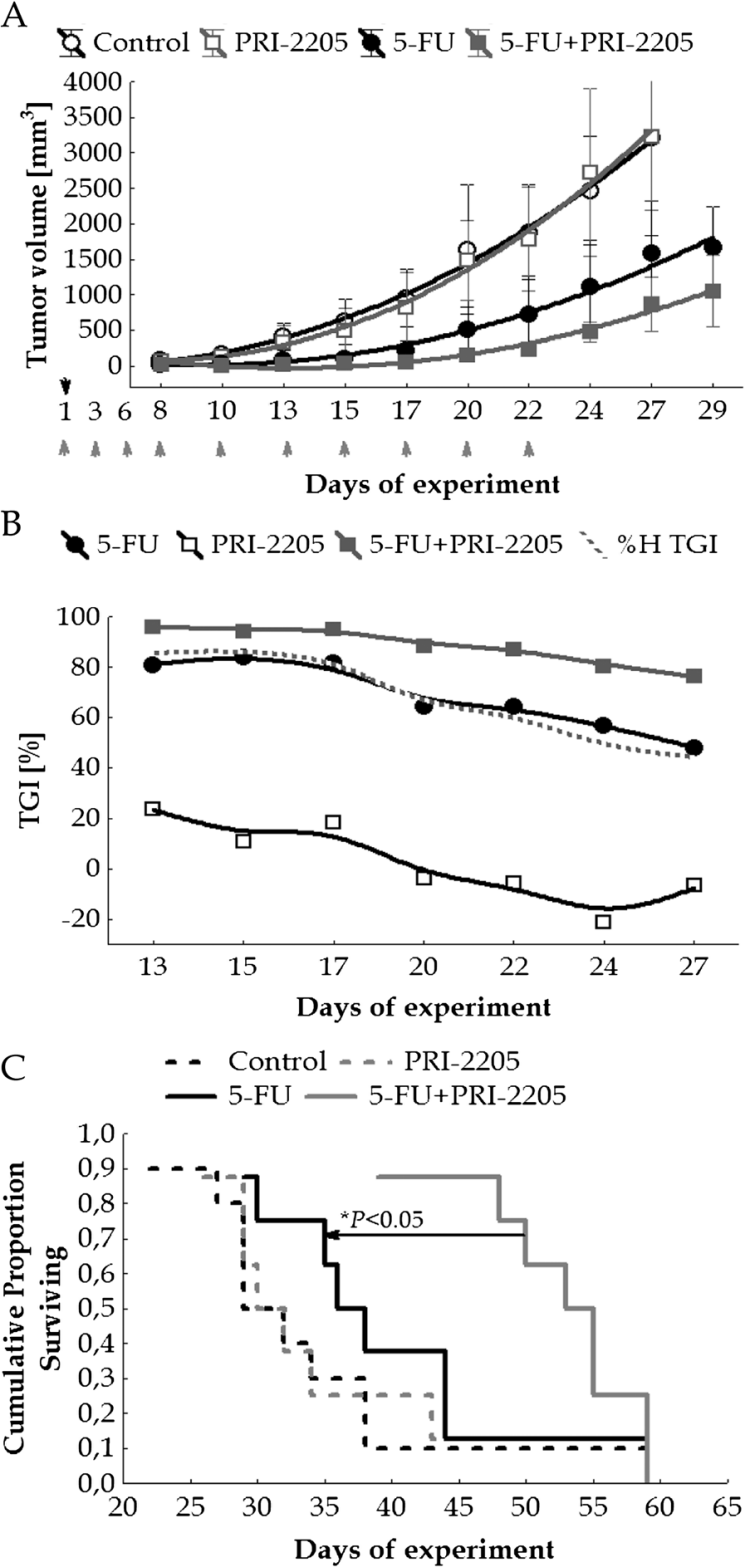
**The influence of PRI-2205 on the antitumor activity of 5-FU in MC38 colon cancer.** PRI-2205 was administered at a dose of 10 μg/kg/day, s.c., three times a week, from day 1 to 22, 5-FU at a dose of 75 mg/kg/day, i.p., on the first day. Black arrow indicate day of 5-FU administration, gray arrows – days of PRI-2205 administration. **A**) Kinetics of tumor growth (mean of tumor volume). *P* < 0.05: 5-FU combined with PRI-2205 in comparison to the control from days 10 to 27 (Kruskal-Wallis ANOVA Multiple Comparisons *P* values (2-tailed) test). **B**) Tumor growth inhibition (TGI) and hypothetical TGI values were calculated from days 13 to 27 of the experiment (median of tumor volume). **C**) Survival analysis of treated and untreated mice. Number of mice: control - 10; in the each treatment group – 8. Statistical analysis was performed for tumor growth inhibition.

The application of PRI-2205 at a dose of 5 μg/kg/day did not improve the therapeutic effect of 5-FU (Table 2, No. 4). In the case of the application of a lower dose (2.5 μg/kg/day), prolonged survival of mice was observed when treated with both agents, but not in a significant manner. Moreover, the interaction between both agents in the case of TGI analysis indicated antagonism (Table 2, No. 5). The results indicate that the most effective dose for PRI-2205 is 10 μg/kg/day. We performed three experiments, in which the same dose of PRI-2205 (10 μg/kg/day) and also varied doses and treatment schedules of 5-FU (50, 75 or 150 mg/kg/day) were applied. A synergistic effect was observed in the case of TGI as well as ILS, independent of the dose of 5-FU (Table 2, No. 1, 2, 3; Figure 2A, B, C). However, the combined treatment with the highest dose of 5-FU (150 mg/kg/day) caused high toxicity (Table 2, No. 3).

*Analog PRI-2191 or PRI-2205 was administered s.c., five times a week.* Both vitamin analogs slightly slowed down MC38 tumor growth in mice treated with 5-FU and caused a subadditive or synergistic effect at the end of the experiment on the 25th day, respectively (Table 2). Furthermore, only analog PRI-2205 prolonged the survival of mice treated with 5-FU. This combined treatment was not toxic (Table 3).

**Table 3:** The effect of vitamin D analogs alone or in combination with 5-FU on tumor growth, life span and body weight of mice bearing subcutaneously MC38 colon cancer

**Analog (AN)**	**5-FU**	**AN**	**TGI [%] on the 25th day of the experiment**					**ILS [%]**					**Maximal decrease**		
													**in body weight [%]**		
			**5-FU**	**AN**	**5-FU + AN**	**%H TGI**	**Effect**	**5-FU**	**AN**	**5-FU + AN**	**%H ILS**	**Effect**	**5-FU**	**AN**	**5-FU + AN**
PRI-2191	100 mg/kg	0.2 μg/kg	69	42	73	82	Subadditive	63	4	67	64	No effect	4	7	4
PRI-2205	100 mg/kg	20 μg/kg	69	-16	72	64	Synergism	63	0	83	62	Synergism	4	5	1

Vitamin D analog was administered subcutaneously, five times a week.

*AN* Analog of vitamin D, *TGI* Tumor growth inhibition, %*H TGI* Hypothetical tumor growth inhibition, *ILS* Increase in life span, %*H ILS* Hypothetical increase in life span, Number of mice: control - 9; PRI-2191 – 7, PRI-2205: 7, 5-FU: 5, 5-FU + PRI-2205: 6, 5-FU + PRI-2191 – 7.

Mice bearing s.c. MC38 colon cancer cells were i.v. injected with 5-FU at a dose of 100 mg/kg/day on days: 2 and 17 and/or vitamin D analogs: PRI-2191 at a dose of 0.2 μg/kg/day or PRI-2205 at a dose of 20 μg/kg/day. Both analogs were administrated s.c., five times a week from days: 9 to 25.

*Analog PRI-2191 or PRI-2205 administered p.o., three times a week.* Combined treatment with PRI-2191 decreased tumor growth by 81%, while 5-FU alone by 72% (P > 0.05). The interaction between both agents indicated synergy. The maximal decrease in body weight did not exceed 15% (Table 4). However, analog PRI-2205 administered orally did not improve the therapeutic effect of 5-FU (Table 4).

**Table 4:** The effect of vitamin D analog (in the most effective dose, administered orally) alone or in combination with 5-FU on tumor growth and body weight of mice bearing subcutaneously MC38 colon cancer

**Analog (AN)**	**5-FU**	**AN**	**TGI [%] on the 21st day of the experiment**					**Maximal decrease**		
								**in body weight [%]**		
			**5-FU**	**AN**	**5-FU + AN**	**%H TGI**	**Effect**	**5-FU**	**AN**	**5-FU + AN**
PRI-2191	100 mg/kg	1 μg/kg	72	12	81	75	Synergism	2	5	15
PRI-2205	100 mg/kg	10 μg/kg	72	6	73	73	No effect	2	5	5

Vitamin D analog was administered orally (p.o.), three times a week.

*AN* Analog of vitamin D, *TGI* Tumor growth inhibition; *%H TGI*, Hypothetical tumor growth inhibition; Number of mice in each group - 8.

Mice bearing s.c. MC38 colon cancer cells were i.v. injected with 5-FU at a dose of 100 mg/kg/day from days 9 to 23 and/or vitamin D analogs: PRI-2191 at a dose of 1 μg/kg/day or PRI-2205 at a dose of 10 μg/kg/day. Both analogs were administrated p.o., three times a week from days 12 to 28.

### The effect of combined therapy with 5-FU and PRI-2191 or PRI-2205 on MC38 colon tumor growth - implanted orthopically

**Figure 3:**
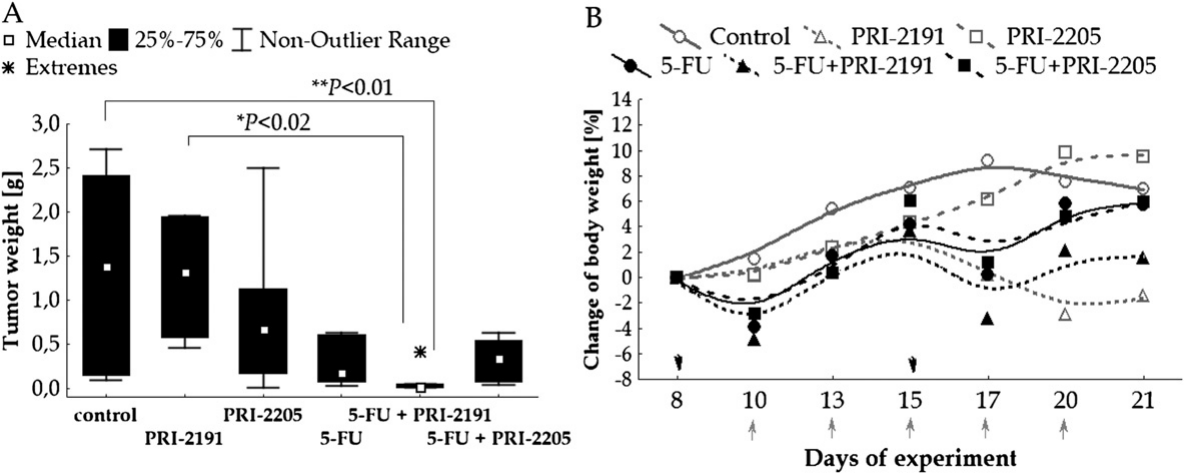
**The effect of PRI-2191 or PRI-2205 alone or in combination with 5-FU on MC38 tumors transplanted ortotopically. A**) Intestinal tumor weight. **B**) Body weight of mice during treatment. Mice bearing MC38 tumors implanted i.i. were i.v. injected with 5-FU at a dose of 100 mg/kg/day on days 8 and 15 and vitamin D analogs: PRI-2191 at a dose of 1 μg/kg/day or PRI-2205 at a dose of 10 μg/kg/day. Both analogs were administrated s.c., three times a week from days 10 to 20. Mice were sacrificed on the 22nd day after i.i. transplantation, tumors were weighed and blood was collected. Black arrows indicate the days of 5-FU administration, gray – days of vitamin D analogs administration. Number of mice: control - 7; PRI-2191 – 4, PRI-2205: 6, 5-FU: 6, 5-FU + PRI-2191 – 7; 5-FU + PRI-2205: 6; ^**^P < 0.01 – 5-FU + PRI-2191 compared to control; ^*^P < 0.05 – 5-FU + PRI-2191 compared to PRI-2191.

As described above, the most effective treatment schedule and dose of both vitamin D analogs is s.c. injection, three times a week and at a dose of 1 μg/kg/day for PRI-2191 and 10 μg/kg/day for PRI-2205. In this experiment, we evaluated the influence of vitamin D analogs (in the most effective dose and treatment schedule) on the antitumor activity of 5-FU in the treatment of mice bearing MC38 colon tumors transplanted orthopically. On the 22nd day of the experiment, intestinal tumors were isolated and weighed. In mice treated with 5-FU alone, the reduction of tumor weight was 79% when compared to untreated mice (P > 0.05). Combined treatment with 5-FU and PRI-2191 significantly (94%) reduced the intestinal tumor growth. The interaction between these two compounds indicated synergy (Figure 3A, Table 4). The kinetics of body weight changes in treated and untreated mice is illustrated in Figure 3B. The combined treatment with 5-FU and PRI-2191 did not cause toxicity. The maximal decrease in body weight did not exceed 5% (Figure 3B). The analog PRI-2205 did not improve the antitumor effect of 5-FU (Figure 3A).

On the other hand, 5-FU applied alone increased the number of blood leukocytes and platelets in relation to the control mice. In mice treated with 5-FU and PRI-2191 or PRI-2205, the tendency for a decrease in the number of blood leukocytes was noted in comparison with 5-FU alone, but there was no change compared to control. In terms of the number of platelets, no difference between 5-FU alone and in combined treatment was observed (Table 5).

**Table 5:** The influence of PRI-2191 or PRI-2205 alone or in combination with 5-FU on intestinal tumor growth, leukocytes and platelets in the blood samples from mice with MC38 colon cancer

**Group**	**Tumor weight**				**Leukocytes**	**Platelets**	**N**
	**Mean ± SD [g]**	**TGI [%]**	**%H TGI**	**Effect**	**Mean ± SD [10**^**3**^**/μL]**	**Mean ± SD [10**^**3**^**/μL]**	
Control	1.4 ± 1.06				7.7 ± 3.4	684 ± 309	7
PRI-2191	1.26 ± 0.80	4			7.2 ± 1.4	835 ± 170	4
PRI-2205	0.86 ± 0.93	35			8.7 ± 2.3	887 ± 67	6
5-FU	0.28 ± 0.27	79			15.5 ± 5.7	1186 ± 207^**c^	6
5-FU + PRI-2191	0.08 ±0.15^**a,*b^	94	80	Synergism	8.6 ± 3.0	1159 ± 254^**c^	7
5-FU + PRI-2205	0.32 ± 0.25	75	86	Antagonism	7.6 ± 4.1	1046 ± 320	6

*TGI*, Tumor growth inhibition; %*H TGI*, Hypothetical tumor growth inhibition; *N*, Number of mice in group; *SD*, Standard deviation.

^**a^*P* < 0.01 as compared to control; ^*b^*P* < 0.05 as compared to PRI-2191: Kruskal-Wallis ANOVA (Multiple Comparisons p values (2-tailed)); ^**c^*P* < 0.01 as compared to control: Tukey HSD.

Mice bearing MC38 tumors implanted i.i. were i.v. injected with 5-FU at a dose of 100 mg/kg/day on days: 8, 15 and/or vitamin D analogs: PRI-2191 at a dose of 1 μg/kg/day or PRI-2205 at a dose of 10 μg/kg/day. Both analogs were administrated s.c., three times a week from days 10 to 20. Mice were sacrificed on day 22 after i.i. transplantation, tumors were weighed and blood was collected.

### The prolongation of 5-FU antitumor activity by PRI-2191 or PRI-2205

**Figure 4:**
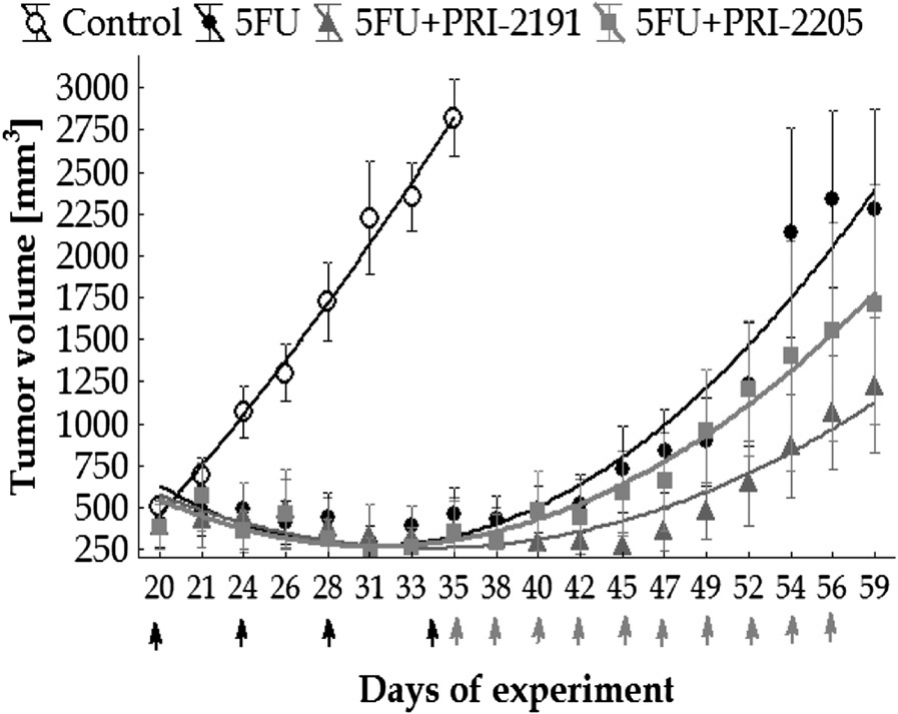
**The anticancer activity of vitamin D analogs injected after the completion of 5-FU administration in the treatment of mice bearing MC38 colon cancer.** The kinetics of tumor growth (mean tumor volume). Mice bearing s.c. MC38 colon cancer cells were i.v. injected with 5-FU at a dose of 100 mg/kg/day from days 20 to 34. When 5-FU reduced tumor volume by about 90% in comparison to the control group, the administration of analogs was initiated (after administration of the cytostatic drug ended). Both analogs were administrated s.c., three times a week from days 35 to 56, PRI-2191 at a dose of 1 μg/kg/day and PRI-2205 at a dose of 10 μg/kg/day. Black arrows indicate the days of 5-FU administration, gray – days of vitamin D analog administration. Number of mice in the control group – 8 and in each treated group – 6. Statistical analysis: Kruskal-Wallis ANOVA Multiple Comparisons *P* values (2-tailed) test. *P* < 0.05 compared to control group: 5-FU from days 26 to 33, 5-FU + PRI-2191 as well as 5-FU + PRI-2205 from days 28 to 35. On day 35 the control group was sacrificed because of large size of tumors.

The application of analogs was started from the 35th day of the experiment after the end of 5-FU administration (i.e. when cytostatic reduced tumor volume by 90% in relation to control untreated mice). As shown in Figure 4, after treatment with 5-FU alone ended a rapid increase in tumor growth was noted. However, in the case of combined treatment, we observed that the application of vitamin D analogs prolonged the antitumor activity of 5-FU. Both analogs delayed tumor growth as well as prolonged the survival of mice in comparison with 5-FU given alone (Figure 4, Table 6). Better results were observed in the case of PRI-2191 than PRI-2205. To compare the time needed to reach 1 cm^3^ tumor of volume in the treated groups in relation to the control group, tumor growth delay (TGD) was calculated. As shown in Table 6, administration of 5-FU alone delayed tumor growth by 23 days compared to untreated mice, in the case of combined treatment by 27 days using PRI-2205 and 32 days using PRI-2191. Moreover, the 105% prolongation of the survival of mice treated with PRI-2191 after 5-FU was noted, while prolongation amounted by 76% with 5-FU alone (Table 6). In addition, no toxicity for 5-FU and combined treatment was observed (data not shown).

**Table 6:** Tumor growth delay values for each group and survival analysis of mice

**Group**	**The time needed for tumors to reach**			**ILS [%]**	**N**
	**the volume of 1 cm**^**3**^				
	**TGD [day]**	**ΔTGD**_**control**_	**ΔTGD**_**cytostatic**_		
		**[day]**	**[day]**		
Control	24	---		---	8
5-FU	47	23		76^**^	6
5-FU + PRI-2205	50	27	3	97	6
5-FU + PRI-2191	56	32	9	105^**^	6

*N* Number of mice in group, *TGD* Tumor growth delay, ΔTGD_control_ = TGD _treated group_–TGD_control_, ΔTGD_cytostatic_ = TGD_treated group_–TGD_cytostatic_, *ILS* increase in life span, ^**^P < 0.01 as compared to control.

Mice bearing s.c. MC38 colon cancer cells were i.v. injected with 5-FU at a dose of 100 mg/kg/day from days 20 to 34. When 5-FU reduced tumor volume by about 90% in comparison to the control group, the administration of analogs was initiated (after administration of the cytostatic drug ended). Both analogs were administrated s.c., three times a week from days 35 to 56, analog PRI-2191 at a dose of 1 μg/kg/day and PRI-2205 at a dose of 10 μg/kg/day.

### The antimetastatic effect of combined therapy with 5-FU and PRI-2191 or PRI-2205

**Figure 5:**
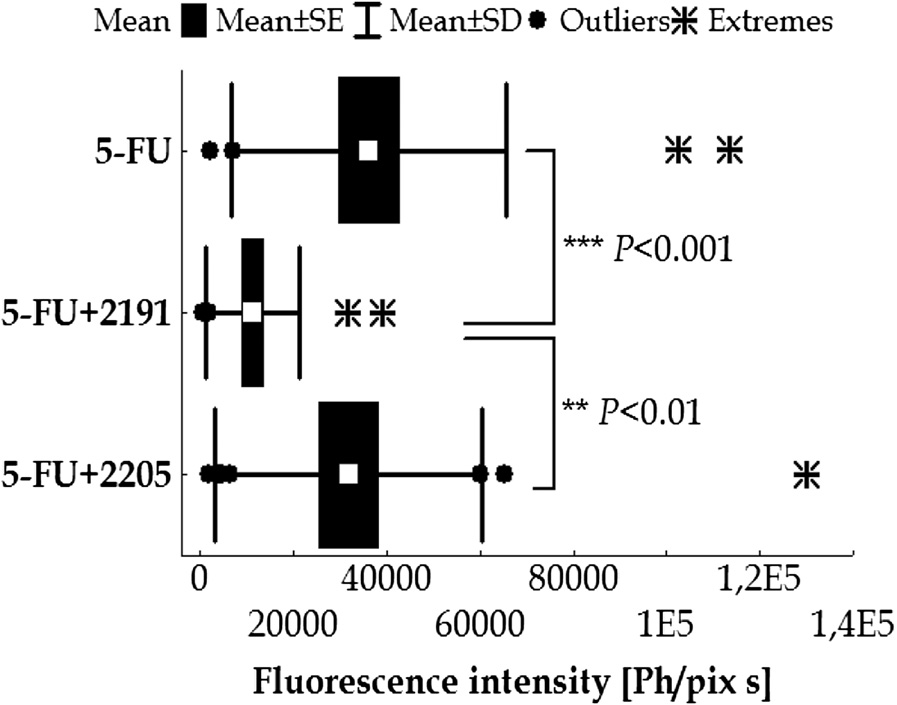
**The metastasis of MC38/EGFP colon cancer cells to lymph nodes in mice treated with 5-FU and PRI-2191 or PRI-2205.** Mice bearing s.c. MC38/EGFP colon cancer cells were i.p. treated with 5-FU at a dose of 75 mg/kg/day from days 19 to 29 alone and in combination with vitamin D analog PRI-2191 at a dose of 1 μg/kg/day or PRI-2205 at a dose of 10 μg/kg/day. Both analogs were administrated s.c., three times a week from days 19 to 56. The mean fluorescence intensity value of the green fluorescent protein which is directly proportional to the number of MC38/EGFP cells in lymph nodes isolated from mice on the 59th day of the experiment was calculated. The number of mice in each group – 7. Statistical analysis was performed using the Kruskal-Wallis ANOVA Multiple Comparisons *P* values (2-tailed) test. ^***^P < 0.001 – 5-FU + PRI-2191 compared to 5-FU; ^**^P < 0.01 – 5-FU + PRI-2191 compared to 5-FU + PRI-2205.

Next, we investigated the influence of vitamin D analogs on regional lymph node metastasis formation in the treatment of mice bearing MC38/EGFP colon cancer cells. The combined treatment using 5-FU and PRI-2191 significantly decreased colon cancer metastasis to lymph nodes compared to 5-FU applied alone (Figure 5). The application of 5-FU and PRI-2191 decreased metastasis up to 5 times in relation to untreated mice, while for 5-FU alone it was up to 2 times (data not shown). Moreover, an 80% inhibition of tumor growth was observed on the 49th day of experiment for combined treatment with PRI-2191 and 62% for 5-FU alone. The interaction between these two agents indicated synergy. No toxicity for 5-FU either alone or in combined treatment was observed (data not shown). However, analog PRI-2205 had no effect on the antimetastatic activity of 5-FU (Figure 5).

### The effect of combined therapy with capecitabine and PRI-2191 or PRI-2205 on MC38 colon tumor growth - implanted subcutaneously

**Figure 6:**
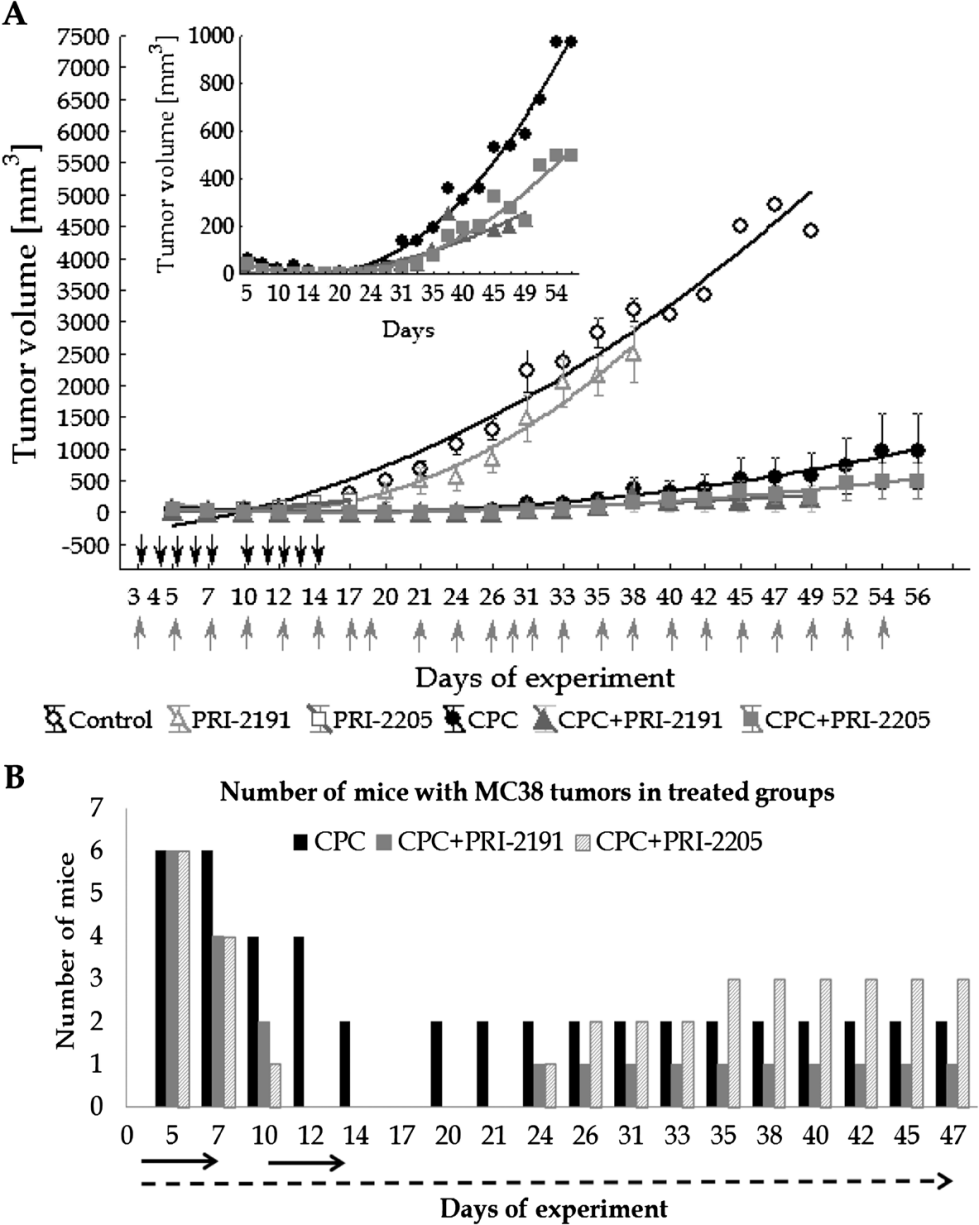
**MC38 tumor growth in mice treated with capecitabine (CPC) alone and in combination with vitamin D analogs. A**) The kinetics of tumor growth. **B**) The number of mice with tumors in treated groups during experiment. CPC was administered p.o. at a dose of 450 mg/kg/day and/or vitamin D analogs: PRI-2191 at a dose of 1 μg/kg/day and PRI-2205 at a dose of 10 μg/kg/day, both were administrated s.c., three times a week. Black arrows indicate the days of CPC administration and gray – days of vitamin D analog administration. The number of mice: control - 8; PRI-2191 – 4, PRI-2205: 3, 5-FU: 6, 5-FU + PRI-2191 – 6; 5-FU + PRI-2205: 6. Statistical analysis: Kruskal-Wallis ANOVA Multiple Comparisons *P* values (2-tailed) test. *P* < 0.05 as compared to the control: CPC from days 14 to 35; PRI-2191 in combined treatment with CPC from days 10 to 35; PRI-2205 in combined treatment with CPC from days 10 to 33.

Capecitabine used at a dose of 450 mg/kg/day, significantly retarded MC38 tumor growth from day 14 to 35 as compared to the control group (*P* < 0.05). In a combined treatment strategy, PRI-2191 and PRI-2205 improved the effect of capecitabine (Figure 6A). Both analogs applied in combined treatment with capecitabine significantly retarded the growth of tumors (*P* < 0.05 from days 10 to 35 – PRI-2191, and from days 10 to 33 – PRI-2205). On day 17 of the experiment a complete regression of tumor growth was observed in mice treated with capecitabine and this lasted until day 20. Both analogs shortened the time for the regression of capecitabine treated tumors: tumors disappeared on day 12, and occurred on day 24 of the experiment (Figure 6B). Maximal body weight decrease reaching 5% was observed in mice treated with capecitabine alone or combined with PRI-2191 (data not shown).

#### *Calcemic activity of combined treatment*

The results of the serum calcium level evaluation after s.c. injection of PRI-2191 or PRI-2205 (2 and 10 μg/kg/day), estimated on 16 day after the first injection, are illustrated in Table 7. Calcium level in serum of mice treated with PRI-2191 alone or combined with 5-FU was significantly higher than this in untreated mice. However, administration of 10 μg/kg/day of PRI-2205 did not affect calcium level.

**Table 7:** Serum calcium level on day 16 after first administration of PRI-2191 or PRI-2205 and 5-FU

	**Control**	**5-FU**	**PRI-2191**	**PRI-2205**	**PRI-2191 + 5-FU**	**PRI-2205 + 5-FU**
N	5	5	4	5	6	4
Calcium level [mEq/L]	4.97 ± 0.08	5.02 ± 0.09	5.61 ± 0.2*	5.04 ± 0.16	5.57 ± 0.16*	5.15 ± 0.06

*Kruskal-Wallis test for multiple comparisons; *P* < 0.05 as compared to control.

*N* Number of mice.

#### *In vitro studies on colon cancer cell lines*

In these studies we used two reference compounds, namely calcitriol and calcipotriol (PRI-2201). As shown in Table 6, most sensitive to the antiproliferative activity of vitamin D compounds among the cell lines tested is human colon cancer HT-29. Moreover, the potentiation for 5-FU antiproliferative effect by vitamin D compounds was clearly observed on this cell line (Table 8). Other cell lines were less sensitive or almost non-sensitive to proliferation inhibition by vitamin D analogs. However, when MC38/0 cells were incubated with low concentrations of 5-FU, proliferation was inhibited by 9% and raised to 41% when PRI-2205 was included (Table 8). On the basis of these studies, we chose HT-29 and MC38/0 cells for further in vitro studies. All in vitro studies on the HT-29 cells described below were conducted in the same concentrations of compounds, such as were used in antiproliferative activity tests.

**Table 8:** The antiproliferative effect of vitamin D analogs with 5-FU on human and mouse colon cancer cells

**Vitamin D compounds**	**Colon cancer cell line [%]**								
	**HT-29**			**LoVo**			**MC38/0**		
		**+ 5-FU**	**%H**		**+ 5-FU**	**%H**		**+ 5-FU**	**%H**
**-**		**8 ± 7**	**-**		**10 ± 5**	**-**		**9 ± 7**	**-**
**Calcitriol**	32 ± 5	49^ab^ ± 6	*37*	0	25^a^ ± 11	*10*	5 ± 8	24 ± 2	*14*
**PRI-2191**	28 ± 4	46^ab^ ± 5	*34*	0	14 ± 7	*10*	0	12 ± 4	*9*
**PRI-2201**	29 ± 5	48^ab^ ± 6	*35*	0	14 ± 7	*10*	0	16 ± 7	*9*
**PRI-2205**	8 ± 3	24^ab^ ± 8	*16*	9 ± 6	24 ± 6	*18*	13 ± 10	41^ab^ ± 10	*17*

[%] – proliferation inhibition.

%H = 100-[(100-E for cytostatic) × (100-E for calcitriol analog) /100], where E was the percentage of proliferation inhibition.

On HT-29 and LoVo cells 5-FU was used in concentrations of 0.1 μg/ml and on MC38/0 – 0.01 μg/ml. Vitamin analogs were used in concentrations of 100 nM.

^a^*P* < 0.05 as compared to 5-FU; ^ab^*P* < 0.05 as compared to calcitriol or vitamin D analog.

**Figure 7:**
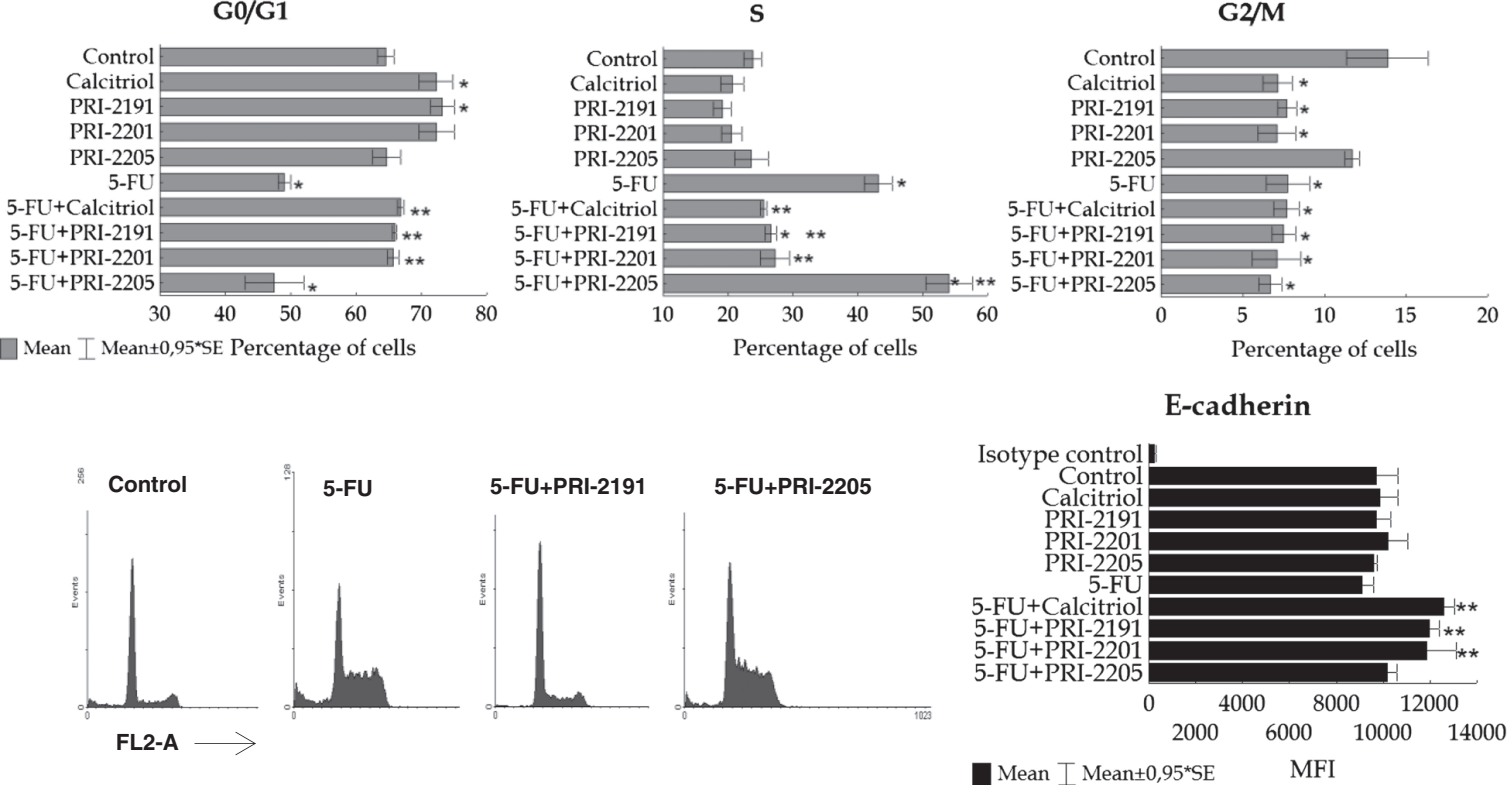
**Cell cycle distribution and E-cadherin expression on HT-29 cells.** Human colon cancer cells HT-29 were incubated with calcitriol or its analogs alone and combined with 5-FU for 120 h in the case of the cell cycle assay and 48 h for E-cadherin expression analysis. Calcitriol and its analogs were used in concentrations of 100 nM, 5-FU was used at a concentration of 0.1 μg/ml both for cell cycle and E-cadherin expression analysis. * *P* < 0.05 as compared to control, ** *P* < 0.05 as compared to 5-FU (Mann–Whitney U test).

The evaluation of the cell cycle is presented in Figure 7. As shown in the upper panel of Figure 7, calcitriol, PRI-2201, as well as PRI-2191 tend to increase the percentage of cells in the G_0_/G_1_ stage, whereas PRI-2205 did not affect the cells in this phase. This was inversely correlated with the percentage of cells in the G_2_/M phase. Namely, the three mentioned compounds decreased the cells in G_2_/M, but PRI-2205 increased them. Analyzing the cells treated with 5-FU, we observed that the cells treated with this agent alone are cumulated in the S phase and decreased in the G_0_/G_1_ and G_2_/M phases, when compared to control. Parallel incubation of cells with calcitriol, PRI-2201 and PRI-2191, but not by PRI-2205, increased the percentage of cells in G_0_/G_1_ as compared to 5-FU alone. Moreover, the number of cells in the S and G_2_/M phases was decreased by these three compounds as compared to 5-FU. The action of PRI-2205 was different; this analog either did not influence or only slightly decreased the cells in G_0_/G_1_ and G_2_/M, but increased them in the S phase as compared to 5-FU alone. The percentage of death cells (subG_1_) did not on average exceed 10 percent (data not shown). Moreover, in this lower panel of Figure 7, representative histograms are presented for control cells, as well as those incubated with 5-FU alone or combined with PRI-2191 and PRI-2205.

In the series of in vitro studies we also analyzed the expression of E-cadherin on HT-29 cells. A statistically significant increase in this adhesion molecule was observed on cells incubated with 5-FU used in combination with calcitriol, PRI-2201 and PRI-2191 (Figure 7, lower panel).

**Figure 8:**
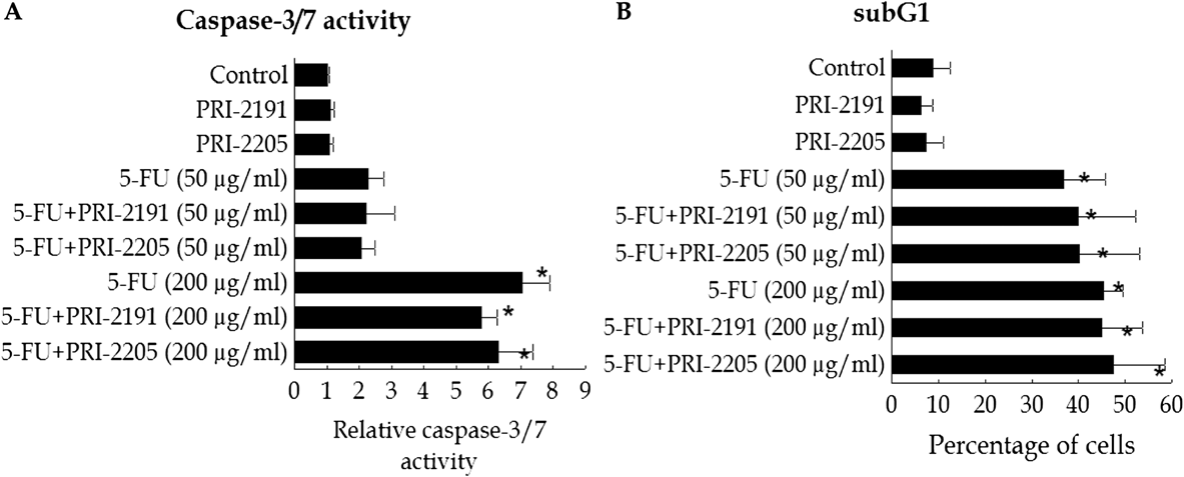
**Apoptosis of MC38/0 cells.** Active caspase-3/7 (**A**) and subG_1_ stage (**B**) of MC38/0 murine colon cancer cells after 48 h incubation with vitamin D analogs and/or 5-FU. Vitamin analogs were used in concentrations of 100 nM. 5-FU was used at concentrations of 50 μg/ml and 200 μg/ml. Asterisks indicate statistically significant results as compared to the control Tukey HSD test for active caspase-3/7 and the Mann–Whitney U test for death cell (subG_1_) analysis.

Using higher concentrations of 5-FU, we also analyzed the subG_1_ population and the activity of caspase-3/7 in MC38/0 cells incubated with or without vitamin D analogs (Figure 8 A and B). A statistically significant increase in the percentage of cells in the subG_1_ stage was observed independently of the presence of vitamin D compounds, when cells were incubated with 50 or 200 μg/ml of 5-FU. However, a tendency to increase the percentage of cells was observed, when the cells were incubated with PRI-2205 and 5-FU (Figure 8B). Under the same experimental conditions, the activity of caspase-3/7 was evaluated. A statistically significant increase in enzyme activity was observed in cells treated with 5-FU at a dose of 200 μg/ml alone or with vitamin D analogs. The activity of caspase-3/7 was slightly diminished by both analogs as compared to 5-FU alone (Figure 8A).

## Discussion

In the present study we demonstrate, for the first time in an *in vivo* model, the biological effect of combined therapy against MC38 mouse colon cancer using 5-FU along with vitamin D analogs (PRI-2191 - tacalcitol and PRI-2205 – 5,6-trans-isomer of calcipotriol)*.*

**Figure 9:**
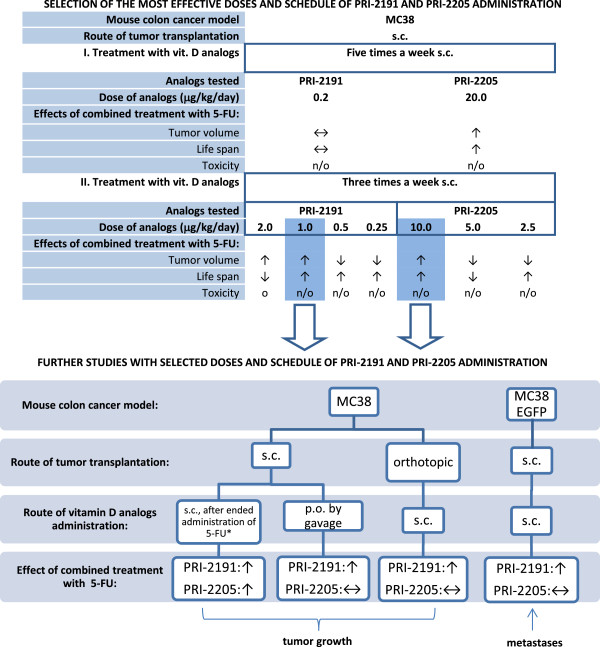
**Work-flow of *in vivo *studies on combined treatment with vitamin D analogs and 5-FU.** ↑ synergistic; ↓ antagonism; ↔ additive or no effect; o – observed; n/o – not observed; * - only in this experiment vitamin D analogs was administered after ended administration of 5-FU, in remaining experiments – simultaneously, as described in methods section.

We observed that PRI-2191, as well as PRI-2205 analog, significantly enhanced the antitumor activity of 5-FU, but these results depend on the treatment regimen. We conclude that the most effective treatment scheme and dose of both vitamin D analogs is when they are injected s.c., three times a week and at a dose of 1 μg/kg/day for PRI-2191 and 10 μg/kg/day for PRI-2205. Work-flow of *in vivo* studies on combined treatment with vitamin D analogs and 5-FU is summarized in Figure 9. A similar administration schedule was applied for vitamin D active form in the first phase of clinical trials. Patients with advanced malignancy received calcitriol s.c. every other day. It has been shown that the intermittent schedule and s.c. injection allow the application of calcitriol at doses up to 4–5 fold higher (with tolerable toxicity) than is the case with oral application. However, calcitriol alone did not show significant antitumor activity [10].

Applying this optimal schedule of combined therapy, we observed a significant decrease of tumor growth, and also a prolongation of survival time of mice in comparison with 5-FU given alone. Most recently published epidemiological studies have shown that diagnosis in the summer and autumn months has been associated with better survival. Authors have suggested that exposure to sunlight and the subsequent higher levels of skin vitamin D synthesis at the time of diagnosis or treatment might be the basis of the patients’ improved survival [28].

Our combined treatment regimen was also successfully applied in the orthotopic model of colon cancer; thus, in an environment similar to natural. Moreover, the vitamin D analogs applied following the administration completion of 5-FU could prolong the antitumor activity of this drug. However, better results were in general observed for PRI-2191 than PRI-2205. Our *in vitro* studies on HT-29 colon cancer cells showed that PRI-2191 acts similarly to calcitriol or PRI-2201 (calcipotriol), whereas the mode of action of PRI-2205 differs. Also, our previous studies showed that PRI-2205 appeared to be less potent in the induction of cancer cell differentiation *in vitro* as compared to calcitriol or PRI-2191 [12,20]. Moreover, this is in accordance with the results of ERK1/2 phosphorylation from our previous *in vivo* studies on HT-29 tumors, where analog PRI-2191 used alone increased the level of p-ERK1/2, which is not observed in tumors from mice treated with PRI-2205 [20].

Thymidylate synthase (TS) is an important target for 5-FU. Improved 5-FU activity might be achieved, e.g., by increasing and prolonging TS inhibition and prevention of TS induction [29]. Our *in vitro* studies on HT-29 cells showed that PRI-2191 – the more active analog – used along with 5-FU caused accumulation of cells in the G_0_/G_1_ cell cycle phase with a parallel decreasing of cells in the S stage. It is possible that this action of PRI-2191 is responsible, in part, for an increased sensitivity of colon cancer to 5-FU, because the expression of TS is the highest during S phase progression and decreases when the cells do not proliferate [29]. A different mechanism was observed in the case of analog PRI-2205 applied along with 5-FU. PRI-2205 combined with 5-FU significantly increases cell percentage in the S cell cycle phase compared to 5-FU applied alone, which indicates that one of the direct mechanisms of combination therapy may in part be attributable to the synergistic effect of both compounds. *Liu et al.* reported that calcitriol suppressed the expression of TS in colon cancer cells and promoted a cytotoxic response to 5-FU [24]. Our further studies on MC38/0 cells have shown that PRI-2205 indicates a tendency to enhancing cell death induction by 5-FU but in parallel decreases the activity of caspase-3/7 compared to 5-FU, while 5-FU significantly induces its activity. A similar effect was described by *Koren et al.* after simultaneous incubation of HT-29 cells with H_2_O_2_ and calcitriol. The increased cytotoxic effect of combined treatment was shown, but the activity of caspase-3 was decreased by calcitriol compared to H_2_O_2_[30].

The differentiation of colonic epithelial cells may be regulated by extracellular Ca^2+^ and the CaSR. Moreover, the induction of E-cadherin may be an important mechanism underlying the chemopreventive action of Ca^2+^ and calcitriol in colon cancer [31-33]. This is in accordance with our studies, in which PRI-2191, similar to calcitriol, promoted the expression of E-cadherin on HT-29 cells in *in vitro* culture when used along with 5-FU. On the other hand, PRI-2205 lacked this activity. We also observed that 5-FU combined with PRI-2191 causes a significant decrease in colon cancer metastasis to lymph nodes compared to 5-FU applied alone. Published data from clinical studies showed that E-cadherin expression was significantly downregulated in gastrointestinal tumors with distant metastases [34]. Our experiments showed that the antimetastatic potency of PRI-2191 combined with 5-FU is correlated with an increase in E-cadherin expression *in vitro*. On the other hand, a different mechanism of antimetastatic activity may also be considered. Our previous studies showed that the *in vivo* inhibition of mouse Lewis lung (LLC) tumor metastasis after treatment with calcitriol and analog PRI-2191 was a consequence of diminished integrin α_v_β_3_ expression [35].

As we previously showed, both analogs used in *in vivo* studies were less toxic than calcitriol. However, PRI-2191 in therapeutic doses induces a rise in serum calcium levels [19], which is not observed in the case of PRI-2205 [12], these results are confirmed in the combined treatment with 5-FU (Table 7). The slight increase in serum calcium levels by analog PRI-2191 without any evidence of toxicity may favorably affect cell-cell adhesion mediated by E-cadherin which is dependent on adequate calcium levels [31,32].

A number of *in vitro* and *in vivo* studies have shown that calcitriol and its analogs enhance the antiproliferative and antitumor efficacy of multiple chemotherapeutic agents in different cancer models [17,36-39]. It has been demonstrated that calcitriol combined with cisplatin causes a higher tumor growth inhibition than cisplatin alone in the treatment of mice bearing Y-79 human retinoblastoma [40], as well as Lewis lung carcinoma tumors [15]. However, in both studies combined therapy induced significant toxicity. Neither combination used in our studies caused toxicity, expressed as a body weight decrease. Thus, similar to oxaliplatin and irinotecan [20], which are agents used as a components of FOLFOX (5-fluorouracil, folinic acid and oxaliplatin) and FOLFOXIRI (5-fluorouracil/leucovorin, oxaliplatin, and irinotecan) colon cancer treatment protocols in clinics [41], 5-FU could be safely used in combined treatment with vitamin D analogs.

## Conclusion

In conclusion, both vitamin D analogs PRI-2191 and PRI-2205 enhance the antitumor and antimetastatic activity of 5-FU and capecitabine in colon cancer *in vivo*. The combined therapy, in the optimal treatment schedule, induces a synergistic effect and does not show toxicity. Moreover, both analogs prolong the antitumor effect of 5-FU after the completion of drug administration. These effects are correlated with a pro-differentiating potential of analog PRI-2191, whereas analog PRI-2205 acts through a different mechanism, which requires further investigation.

Our data suggest that these vitamin D analogs might be potentially applied to clinical use in order to enhance the anticancer effect of 5-FU and also prolong its activity in patients suffering from colorectal cancer. However, the attention should be paid on such conclusions, because, the relationship between vitamin D and cancer is not easy to interchangeable interpretation. A lot of studies have shown a relationship between vitamin D and cancer, however the direct movement from preclinical to clinical conditions is impossible [42]. The dosage of vitamin D analogs appears to be one of the important reasons of indirect translation of preclinical to clinical results. In our studies, lower doses of PRI-2191 act antagonistically with 5-FU (summarized in Figure 9). In clinical studies, in general, single agent vitamin D has not been directly associated with objective tumor response in phase I–II clinical trials. It is probably the result of the fact, that very few clinical studies have been conducted using doses attaining the maximum tolerated dose. There are also questions, if there is known the maximal tolerated dose for patients [43]. Second important clue, is the heterogeneity of patient tumors, which frequently lead to misinterpretation of the results. The animal studies are conducted on particular histological type of tumor, and some clinical studies showed, that consideration of the histological type of tumors during interpretation of the results may change the conclusions [44]. As it is concluded in many papers analyzing the preclinical and clinical studies on vitamin D compounds, the further studies should be pointed to preclinical analysis of the possible large panel of vitamin D targets and the clinical studies should be better planned to eliminate the known defects of those conducted [42,43].

## Abbreviations

5-FU: 5-Fluorouracil; TGI: Tumor growth inhibition; %H TGI: Hypothetical tumor growth inhibition; ILS: Increase of life span; %H ILS: Hypothetical increase of life span; TGD: Tumor growth delay; i.p: Intraperitoneally; i.v: Intravenously; s.c: Subcutaneously; i.i: Orthotopically; p.o: Orally.

## Competing interests

The authors declare that they have no competing interests.

## Authors’ contribution

Participated in research design: JW, MM, AK. Conducted experiments: JW, MM, MP. Contributed new reagents or analytic tools: AK, MM, MP. Performed data analysis: MM, MP. Wrote or contributed to the writing of the manuscript: JW, MM, AK, MP. All authors read and approved the final manuscript.

## Pre-publication history

The pre-publication history for this paper can be accessed here:

http://www.biomedcentral.com/1471-2407/13/294/prepub
